# Pantheon habitat made from regolith, with a focusing solar reflector

**DOI:** 10.1098/rsta.2020.0142

**Published:** 2021-01-11

**Authors:** Nick Woolf, Roger Angel

**Affiliations:** Steward Observatory, University of Arizona, USA

**Keywords:** Moon, lunar habitat, solar collector, greenhouse, *in situ* resource

## Abstract

We describe a polar Moon base habitat using direct solar energy for construction, food production and atmospheric revitalization. With a growing area as large as 2000 m^2^, it could provide for 40 or more people. The habitat is built like the ancient Roman Pantheon, a stone structure with a top circular oculus, bringing in focused sunlight that is spread out to crops below. The conical, corbelled structure is built from cast regolith blocks, held in compression despite the large internal atmospheric pressure by a regolith overlayer 20–30 m thick. It is sealed on the inside against leaks with thin plastic. A solar mirror concentrator used initially to cast the building blocks is later used to illuminate the habitat through a small pressure window at the oculus. Three years of robotic preparation of the building blocks does not seem excessive for a habitat which can be expected to last for millennia, as has the Treasury of Atreus made by similar dry-stone construction. One goal of returning to the Moon is to demonstrate the practicality of long-term human habitation off the Earth. The off-axis, paraboloidal reflecting mirror is rotated about the vertical polar axis in order to direct horizontal sunlight downward to a focus. In this way, the heavy materials needed from Earth to build and power the habitat are largely limited to the solar concentrator and regolith moving and moulding equipment. By illuminating with a reflector rather than with electricity, the solar collection area is 20 times smaller than would be needed for PV cells.

This article is part of a discussion meeting issue ‘Astronomy from the Moon: the next decades (part 1)’.

## 1. Introduction

A human base on the Moon, and later Mars, should be built ideally from local materials, and be self-sufficient. As little as possible should be imported from Earth because of the high cost of transportation. Here, we consider how a lunar habitat might be built almost entirely from regolith (soil) or rock, with sunlight used directly for food production and revitalization of the atmosphere for both humans and plants.

Previous studies have considered use of *in situ* resources for many purposes [1], many requiring advanced materials processing. Our concept requires minimal manufacturing infrastructure. The structure is made of well-fitting blocks of cast regolith, and covered with a thick layer of lunar regolith to contain a breathable atmosphere and to provide also protection from cosmic ray bombardment. Only a thin, low mass, plastic lining is then needed from Earth to make the structure airtight. By contrast, an inflatable structure built to take 7 ton m^−2^ of atmospheric pressure would require thick material with very high tensile strength [2].

Salisbury [3] finds from experiment that at high light levels a human being could be provided with food on a continuous basis from a controlled environment farm about 13 m^2^ in area. Even with a safety factor of as much as four to allow for other crops that might be less productive or an occasional crop failure, 50 m^2^/person should suffice. Boscheri *et al.* [4] have demonstrated water recycling, air revitalization and food production using selected crops within a semi-closed system. LED grow lights to illuminate these crops would require PV cells of up to 50 times this area, per person.

In a previous study of lunar habitat structural design, Ruess [5] analysed in detail a circular–arched cylindrical structure, providing living quarters of 500 m^2^ for a crew of 12, and safely containing an atmospheric pressure of 70 kPa. Made of aluminium, the structural elements weighed 70–90 tons. Ruess considered also the potential for cast regolith, which has ultimate compressive and tensile strengths about 10 times greater than concrete. He suggested that when cast into structural bricks, blocks or other shapes, it could be used in structures that are dominated by compression. Such bricks would be an ideal material for paving lunar rocket launch sites and constructing debris shields surrounding landing pads.

Other designs, e.g. Ziedler *et al.* [6], have explored the use of inflated plastic modules for short-term lunar use. Such a module was attached to the International Space Station. A limitation with inflatable structures beyond low Earth orbit is that they offer no protection from cosmic rays. A stable, sheltered habitat will be needed on the Moon for long-term use. For the lunar south pole, Vogler [2] has proposed a habitat of inflated modules protected from radiation by a 100 m diameter dome of concrete, made using inflatable technology and covered with a 3 m thick layer of regolith.

## 2. A lunar habitat made from local materials

**Figure 1.**
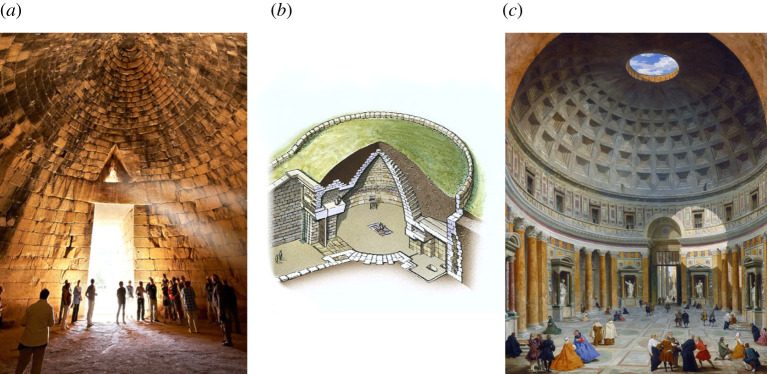
Large enclosed structures still standing after millennia. (*a*) and (*b*) the Treasury of Atreus in Mycenae, and (*c*) the Pantheon in Rome. (Online version in colour.)

Here, we explore the potential for making a habitat from regolith or rock both to provide protection from cosmic rays and to withstand atmospheric pressure. In fact, the largest enclosed structures on Earth until a few hundred years ago were made using locally sourced blocks of stone in compression. We can learn from them. Figure 1*a*,*b* shows the Treasury of Atreus in Mycenae [7]. It is 13.5 m high and 14.5 m in diameter, and it has stood for about 3000 years. Its conical structure of corbelled blocks of stone, with no mortar between, is protected by a covering of earth averaging 4 m in thickness. Figure 1*c* shows the Pantheon, 43 m in diameter, built in AD 120 with a hemispherical dome of concrete blocks [8]. Light enters from above through the 9 m diameter oculus.

We envisage the same type of structure on the Moon, made from regolith cast into well-fitting blocks, and with regolith or rock piled on top to the depth needed to keep the structure always in compression, despite the outward pressure of the internal atmosphere. This structure will be stable in either the pressurized or non-pressurized state. The only tension carrying material needed from Earth is plastic film to seal small gaps between the blocks to prevent air leaking out. The tension in the plastic is proportional to the gap width.

The density of regolith is approximately 1.7 and of cast blocks 3.0. Supposing an atmospheric pressure of 70 kPa (following Ruess) and allowing for the reduced gravity on the Moon, 1/6 that of Earth, a minimum thickness of 25 m of regolith or 15 m of the denser cast blocks is needed.

We assume the habitat will be located near a lunar pole, where there are deposits of water and volatiles to replace atmospheric gas, and sites can be found with near permanent solar illumination. Sunlight will be brought in through a small oculus via a mirror which follows the sun as it moves around the horizon once a month.

In order to get enough sunlight to grow crops through an oculus small enough to be sealed with a transparent pressure window, the light must be focused as it passes through the window before spreading out onto the habitat floor.

## 3. Solar concentrator and regolith block casting

**Figure 2.**
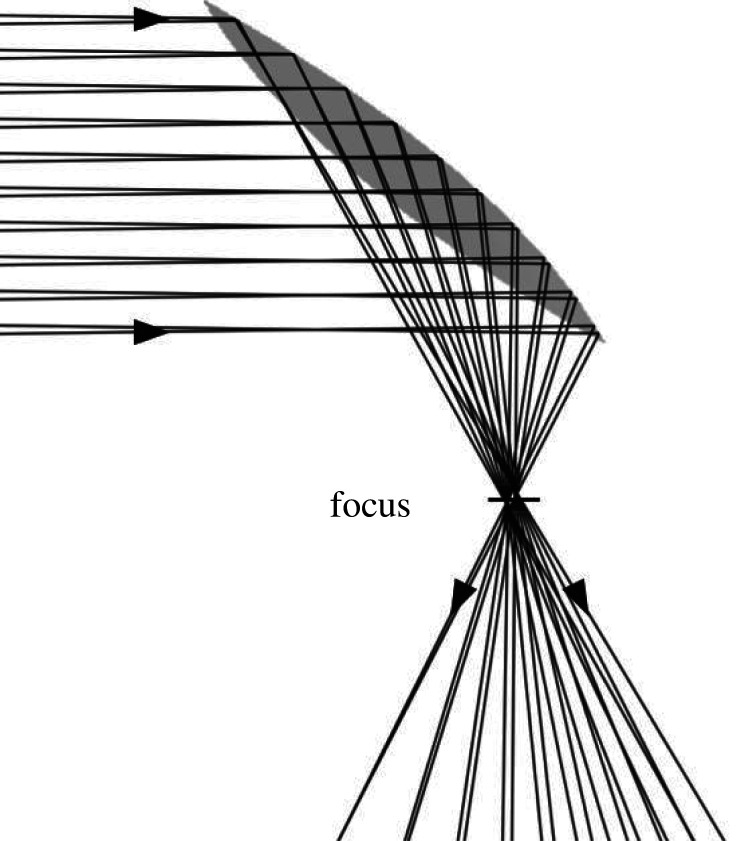
Rotating paraboloidal reflector with rays incident at ±0.75°.

The large mirror above the oculus will be shaped as an off-axis section of a paraboloid, shown in figure 2. We consider here an example in which sunlight is collected over a circular aperture and reflected down to the focus in a 60° cone symmetric about the vertical axis. Ray tracing shows that a perfectly shaped mirror with this geometry would form an image of the 1/2 degree solar disc with a diameter 0.013 that of the entrance aperture. In practice, imperfections in assembly and pointing will lead to a larger focus. We will suppose this to be twice the ideal disc diameter, i.e. 0.026 of the entrance aperture. The concentration of sunlight averaged across this disc is 1400 × 1/(0.026)^2^.

The initial use of the concentrator will be to melt regolith into building blocks. In order to calculate the solar power density at the focus, we assume the mirror is coated as needed for habitat illumination, reflecting the wavelength range 450–650 nm and rejecting the remaining spectrum, to eliminate unnecessary heating when the light is used later to grow crops. Such a reflector may be made with a high efficiency multilayer dielectric coating. The solar power incident in this spectral range is approximately 500 W m^−2^, thus the power density at the focus is 700 kW m^−2^.

Regolith placed at this focus to be melted into blocks may be heated to a temperature limited by the balance of absorbed sunlight energy and thermal losses. Radiative losses will completely dominate, since the blocks will be cast in moulds of ceramic fibre, which is a good thermal insulator, and there is no air or convective cooling. Assuming the melted regolith is effectively black, both to the incoming sunlight and in its thermal emission, the equilibrium temperature that would be reached is 1600°C, determined by black body radiation equalling the input of power of 450 kW m^−2^. The casting temperature for regolith blocks of 1400°C thus should be easily reachable at this focus.

**Figure 3.**
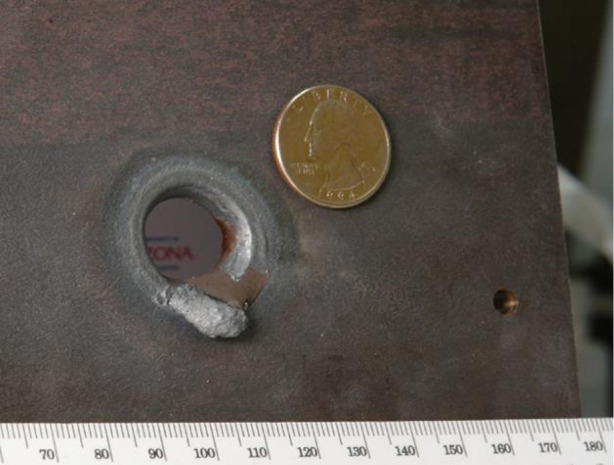
Hole melted in steel by a solar concentrator. (Online version in colour.)

An example of melting at a solar focus is shown in figure 3. The hole shown in a 6 mm thick plate of steel was made in 10 seconds by melting at the focus of a 10 m^2^ paraboloidal solar reflector at the University of Arizona Solar Lab.

The rate at which solar blocks can be made from regolith may be estimated given that the energy required to heat regolith to 1400°C is 360 kWh metric ton^−1^ [5]. Allowing for overall losses of 50%, each square metre of sunlight brought to a focus will provide 0.25 kWh per hour, totalling 1500 kWh year^−1^ at a good polar site with the Sun above the horizon 70% of the time. This is enough to form 4 tons of blocks per year, per square metre of aperture. If the habitat were to be made with blocks in a cone averaging 2 m in thickness, or 6 tons m^−2^, and the solar collector is made with the same area as the habitat floor, and the cone height equals the habitat radius, then the conical surface area is 1.4 times the floor area. Three years of production would be sufficient to produce the blocks. The moulds will be lined with ceramic fibre paper to allow release of the cast blocks and will be shaped to produce the individual block shapes needed, for example, voussoir or trapezoidal. Multiple moulds will be set in a tunnel kiln on a conveyor belt or lazy susan and brought sequentially into position under the solar focus. Once formed, the blocks will be moved on for slow annealing and cooling to avoid thermal stresses. The cooled blocks will be removed, and the mould refilled.

Three years of robotic preparation of the building blocks does not seem excessive for a habitat which can be expected to last for millennia, as has the Treasury of Atreus made by similar dry-stone construction. One goal of returning to the Moon is to demonstrate the practicality of long-term human habitation off the Earth.

PV electric power might be implemented to speed up the rate of block casting. In this case, if the conversion efficiency of AM0 solar radiation into electricity is 20% and panels in fixed orientation convert 1/3 of the available sunlight from all around the horizon, the yield would be an average power of 0.09 kWh h^−1^ m^−2^ for heating, 1/3 of the power per square metre compared to that from the reflector. Additional heating by panels of total area six times that of the mirror could be used to triple production rate, reducing the total casting time to 1 year.

## 4. Concept for a 50 m diameter habitat

### (a) Getting in and distributing the light

The light focused as shown in figure 2 must be brought through the 25 m thick layer of regolith above the structure and then distributed to the crops. The solution chosen here is to transmit the light through a 25 m long light pipe passing through the layer. The most efficient pipe would be a giant fused silica fibre, but this would be too heavy. A 25 m long, 1.3 m diameter fibre would weigh 73 tons. But a hollow pipe can be made with comparably high overall transmission, given highly reflective walls with a multilayer dielectric coating. As an example, such mirrors made commercially to cover the spectral range of wavelengths 400–800 nm, over angles of incidence ranging from 0° to 45°, have a reflectivity greater than 99%. We expect similarly high reflectivity to be obtainable in a coating tailored for incidence angles of 60°–90° and for our narrower spectral range of 450–650 nm.

A typical ray of light passing down the pipe will be reflected approximately 10 times and thus lose in all 10% of its energy. The heating in the pipe wall caused by this loss amounts to 100 kW. Spread over the 100 m^2^ area of the pipe wall, this averages 1 kW m^−2^, no higher power density than sunlight warming the Earth. The same active cooling described below to remove the remaining 90% of the energy absorbed by the floor will be used to maintain the pipe at room temperature, in order to maintain its very high reflectivity through decades of use.

The 1 MW of sunlight exiting the pipe will spread out to illuminate the 50 m diameter growing area at the normal full sunlight intensity. But because the sun will shine 24/7 for much of the time, a means to simulate day and night will be needed, for example, by dividing the light locally between several crops by optical or mechanical means. In lunar winter, there will be periods of several days of continuous darkness each month, even at the most favourable polar sites. Some battery powered LED lighting will likely be necessary for dark periods longer than 24 h, but at a far lower light level, as on very cloudy days on Earth, to minimize battery weight.

### (b) Structural and thermal design

Here we consider a concept for a habitat of specific size, with a growing area 50 m in diameter (2000 m^2^) over living quarters of the same area. This size is informed by the 25 m depth of regolith needed to balance atmospheric pressure, and its 40° angle of repose. Illumination of the growing area at normal solar intensity is accomplished by use of a reflector of the type shown in figure 2, also 50 m in diameter, to collect the same area of sunlight. The focal spot will have a power in the 450–650 nm band of 1 MW, and a diameter of 1.3 m, 0.026 of the 50 m entrance aperture.

**Figure 4.**
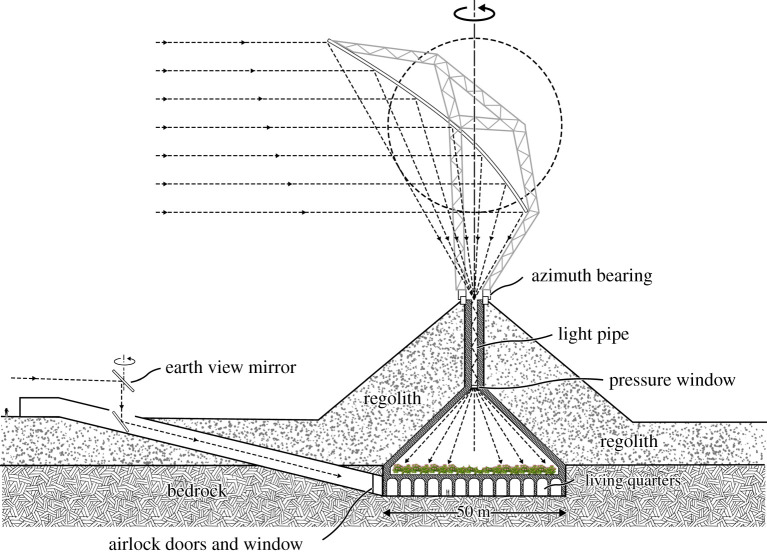
Habitat with 50 m diameter growing area.

In broad brush outline, the structure we consider shown in figure 4 resembles the Atreus Treasury in being built as a cone of blocks under compression from soil above. In fact, the weight of the 25 m of regolith we envisage is no greater than that of the 4 m thickness of soil over the Treasury. The conical corbelled type of structure of the Treasury is favoured because it is adapted to both regolith loading and to inclusion of the oculus opening at the top while still keeping all the blocks in compression. We envisage that detailed finite-element modelling and local testing (allowing for Earth's six times higher gravity) will be used to optimize and validate the design.

The concept for the 50 m diameter habitat shown in figure 4 uses a structural cone 25 m high with a 90° angle. Light is brought in at the apex through a 25 m tall light pipe passing through the regolith overlayer. Light exiting the pipe enters the habitat through a 1.3 m diameter fused quartz pressure window. It is spread out over the full cone angle to illuminate all of the 50 m diameter floor at the standard intensity of sunlight in the wavelength range of visible light.

A solid foundation is needed for the heavy conical compression structure and its burden of piled up regolith. At the lunar south pole, we can expect the regolith to be like that in older highland regions, 10–15 m deep, with a region of blocky and fractured bedrock below [9]. We envision construction beginning with clearing a circular area of regolith down to bedrock. Then enough bedrock will be excavated to create a sturdy flat floor for the habitat, which must be sealed against atmospheric leaks, and to provide a perimeter bedrock wall to resist the outward thrust of the sloping walls of the cone. We envisage that the methods and equipment needed for robotic site preparation and construction of radio and optical telescopes, at the pole and elsewhere, will provide the experience needed in excavation to bedrock.

Living, working and support activity quarters would be built first on the bedrock floor, using cast blocks to make a vaulted ceiling to support the growing area above, and might be extended also around the edge of this area. The access roads sloping down to the base level will later be enclosed as two tunnels like the one shown, to allow safe access to the lunar surface and a direct view of Earth from a window in the living quarters.

The cone is then built up also from cast blocks to the level of the 1.3 m pressure window and regolith brought up to this level. The walls will be sealed on the inside. A possible material would be 0.1 mm thick mylar with a reinforcing scrim, with the plastic sheets fused together at junctions. Punctures will be detectable by the sound of air escaping, and sealed like a tire with sticky patches. The mass of plastic will be about 1 tonne [10]. The block structure is continued up a further 25 m through the regolith cover above the cone as a hollow cylindrical column 5 m in diameter, to support the rotating solar reflector. The inner 1.5 m hole through the column accommodates the light pipe. A flange surrounding the pressure window is set under the column to resist the upward force from atmospheric pressure on the window.

An early example of this type of conical structure may be found in the dome of London's St Paul's Cathedral, where the weight of the 850 ton lantern is taken not by the visible dome, but by a brick cone hidden inside, 0.45 m thick. Our 5 m column of basalt weighs (in the Moon's low gravity) a much lower 245 tons.

To complete the habitat structure, more regolith is piled to the level of the top of the column, in a cone whose 100° angle is set by the angle of repose of regolith. The total volume of regolith to be moved is about 100 000 m^3^. It will be moved into place by a vehicle travelling along a road that spirals up around the regolith cone, just as mining vehicles spiral down into open pit mines. The round-trip distance to bring in a new load of regolith might be around 1 km, thus a vehicle with a carrying capacity of 10 m^3^ travelling at an average speed of 2 m s^−1^ would take 10 min of travel time per trip. Allowing for loading and unloading, it might make perhaps two trips per hour. At this rate, a year of 24/7 operation at a 70% duty cycle would be sufficient to complete regolith piling. The vehicle, a 4 ton vacuum hardened bulldozer equipped with a vibrating blade, would be electrically operated and use about 30 kW. Its batteries will be charged by solar power of approximately 70 kW and could also serve as a mobile power source. Electrical power will be provided by PV panels of some 1000 m^2^ in area, assuming 20% conversion efficiency and fixed orientation resulting in a 30% duty cycle for individual panels.

During construction a dusty atmosphere will be generated, but once construction is complete dust will settle rapidly to a low equilibrium level.

Active cooling of the habitat is needed because the thick layer of regolith will act as strong thermal insulation. We envisage heat transfer fluid being pumped to outside radiators that radiate freely into cold space. They can be painted white and readily shielded from direct sunlight coming from the horizon. A total flow rate of around 10 l s^−1^ will be needed for a water-based transfer fluid cooled 20°C by radiation. The radiator systems will need to have about the same or a little larger area as the 2000 m^2^ of the solar collector and thus must be made with similar attention to lightweighting.

One further thermal design aspect is driven by the need to avoid overheating and damage at the entry to the light pipe. Here we envisage a surround of white ceramic fibre, like the shuttle tiles, which in the event of collector drive failure will reflect away most of the focused energy and radiate away the absorbed component.

### (c) Reflector construction

The 50 m diameter dish reflector and its mount will be brought from Earth. These will be lightweighted as far as possible, consistent with the surface slope accuracy requirement of around 2 mrad to achieve the 1.3 m focal spot targeted. The tracking motion requires 360° rotation about a vertical axis but very little motion in elevation. Gravity is in any case low, and there is no seismic activity on the moon. Under these circumstances, extremely lightweight construction is possible.

We envisage a lightweight dish structure of carbon fibre, with reflector panels of honeycomb sandwich construction. A single rigid structure will link the dish to the 5 m diameter azimuth bearing surrounding the focus. To keep the focal spot centred on the top of the light pipe through the annual changes in solar elevation of ±1.5°, the structure will be actuated to tilt up or down by up to 0.75°. We envisage the same type of lightweight construction as used for the new technology Zeppelin NT [11] whose rigid primary structure, 70 m long and 11 m across, is made of composites and aluminium weighing only 1 ton. On this basis, a total mass of 20 tons might be achievable for the 50 m reflector.

### (d) Alternative of PV power

The question arises as to whether crops might be illuminated in a similar habitat by established technologies, LED grow lights powered by racks of PV panels, rather than by direct sunlight. The weight of lighting equipment to be brought from the Earth would be dominated by that of the panels. If we suppose again that the panels convert AM0 solar radiation into electricity with 20% efficiency, that they are in fixed orientation and convert 1/3 of the total available sunlight from all around the horizon, then to obtain the same 1 MW of growing light, would require 36 000 m^2^ of PV panel area, assuming the LEDs are 30% efficient in converting electricity into light of the required spectrum. If the PV panels used weigh 1 kg m^−2^, their total weight would be 36 tons. Allowing for the weight of racking and LED light fixtures, the total mass for lighting equipment to be brought from Earth would be around 50 tons, significantly more than we project for the rotating mirror system.

A further consideration is PV cell longevity. With no protection from cosmic rays on the Moon, the panels may need to be replaced as frequently as every decade. Another difficulty is that of thermal dissipation. If LEDs are used, then the heat to be transported out of the habitat rises from 1 to 3 MW, and the area of the external radiator increased from 2000 to 6000 m^2^. A three-times larger mass of radiators would need to be brought from Earth.

## 5. Conclusion

From our analysis, it does seem that a substantial habitat for living quarters and agriculture of total area 4000 m^2^ could be built at a lunar pole, sufficient for up to 40 inhabitants and visitors. By building with blocks cast from regolith, the mass of material to be brought from Earth is much reduced. The major items to be brought would be the 2000 m^2^ solar concentrator, radiators, block casting moulds and regolith moving equipment, and pressure window, for a total of perhaps 50 tons or 12 kg m^−2^. This is an order of magnitude less than the 140 kg m^−2^ for the aluminium lunar habitat analysed by Ruess [5].

Further expert analysis including input from architecture, mining and agriculture would be extremely valuable. For example, a computational analysis of the stability of a conical block structure under the regolith and atmospheric loads is needed. Experiments to make test blocks from regolith simulant using concentrated sunlight would be informative. A test facility at Biosphere 2 [12] could demonstrate the use of a solar concentrator and light pipe to grow plants underground. A study to understand how best to apportion the available sunlight across the habitat growing area for the most productive agriculture would be also valuable.

## Data accessibility

This article has no additional data.
